# Targeting Endoplasmic Reticulum and/or Mitochondrial Ca^2+^ Fluxes as Therapeutic Strategy for HCV Infection

**DOI:** 10.3389/fchem.2018.00073

**Published:** 2018-03-21

**Authors:** Rosella Scrima, Claudia Piccoli, Darius Moradpour, Nazzareno Capitanio

**Affiliations:** ^1^Department of Clinical and Experimental Medicine, University of Foggia, Foggia, Italy; ^2^Service of Gastroenterology and Hepatology, Centre Hospitalier Universitaire Vaudois, University of Lausanne, Lausanne, Switzerland

**Keywords:** HCV, mitochondria associated membranes (MAM), calcium channels, viroporin, oxidative phosphorylation, redox signaling

## Abstract

Chronic hepatitis C is characterized by metabolic disorders and by a microenvironment in the liver dominated by oxidative stress, inflammation and regeneration processes that can in the long term lead to liver cirrhosis and hepatocellular carcinoma. Several lines of evidence suggest that mitochondrial dysfunctions play a central role in these processes. However, how these dysfunctions are induced by the virus and whether they play a role in disease progression and neoplastic transformation remains to be determined. Most *in vitro* studies performed so far have shown that several of the hepatitis C virus (HCV) proteins also localize to mitochondria, but the consequences of these interactions on mitochondrial functions remain contradictory and need to be confirmed in the context of productively replicating virus and physiologically relevant *in vitro* and *in vivo* model systems. In the past decade we have been proposing a temporal sequence of events in the HCV-infected cell whereby the primary alteration is localized at the mitochondria-associated ER membranes and causes release of Ca^2+^ from the ER, followed by uptake into mitochondria. This ensues successive mitochondrial dysfunction leading to the generation of reactive oxygen and nitrogen species and a progressive metabolic adaptive response consisting in decreased oxidative phosphorylation and enhanced aerobic glycolysis and lipogenesis. Here we resume the major results provided by our group in the context of HCV-mediated alterations of the cellular inter-compartmental calcium flux homeostasis and present new evidence suggesting targeting of ER and/or mitochondrial calcium transporters as a novel therapeutic strategy.

## Introduction

Liver disease related to HCV infection represents a major health burden worldwide (Di Bisceglie, 1998). Recent estimates suggest around 71 million chronically infected individuals, i.e., 1% of the world population (Cf. WHO Global Hepatitis Report 2017 | http://www.who.int/hepatitis/publications/global-hepatitis-report2017/en/). Approximately 80% of acutely infected individuals develop chronic infection which may progress to cirrhosis in 2–20% after 20 years and in 15–30% after 30 years. Once cirrhosis is established, the risk of hepatocellular carcinoma (HCC) development is 1–5% per year (Alter and Seeff, 2000). Great progress has been achieved in the treatment of chronic hepatitis C in recent years. Currently available directly acting antivirals yield sustained virologic response rates >90%, with very well-tolerated and relatively short treatment regimens (Pawlotsky et al., 2015).

HCV is a positive strand RNA virus belonging to the *Flaviviridae* family and *Hepacivirus* genus discovered in 1989 (Choo et al., 1989). It infects hepatocytes, with the main steps of its life cycle involving: binding to membrane receptors and entry into the cell host; uncoating of the genome from the viral capsid; translation of the viral genome at the ER; replication and assembly; as well as release of the virus particles (Moradpour et al., 2007). Notably, HCV replication and virion assembly takes place in a specialized lipid-enriched cellular compartment of the infected host called membranous web (Dubuisson et al., 2002). It is important to remind that HCV is not cytolytic.

## Mitochondrial oxidative metabolism in HCV infection

The 9.6-kb HCV genome harbors a long open reading frame which is translated into a polyprotein of about 3000 amino acids. This is processed at the level of the ER by cellular and viral proteases to generate 10 proteins. Three of them (structural—core, E1, and E2) contribute to the virus particle, the others (non-structural—p7, NS2, NS3, NS4A, NS4B, NS5A, NS5B) are functional proteins necessary for replication and assembly of the virion (Moradpour and Penin, 2013).

It is amazing how such a limited number of proteins is sufficient to reroute host cell physiology to promote establishment of the infection and viral propagation. An example is given by the capability of HCV to evade innate immunity. This relies in part on the activation of MAVS (mitochondrial antiviral signaling protein) which is anchored on the mitochondrial outer membrane and that after binding of RIG-1 provides a recruiting platform for a number of factors whose activation leads to expression of interferon-β (Seth et al., 2005; West et al., 2011). One of the two viral proteases, the NS3-4A protease, cleaves MAVS, thereby impairing interferon induction (Li et al., 2005; Meylan et al., 2005; Bellecave et al., 2010). The involvement of mitochondria in the viral life cycle is even more pervasive. Indeed, HCV proteins were found to localize at contact sites between the ER and the mitochondrial compartment and move by lateral trafficking to the mitochondrial outer membrane (Mottola et al., 2002; Schwer et al., 2004; Griffin et al., 2005; Kasprzak et al., 2005; Suzuki et al., 2005; Nomura-Takigawa et al., 2006; Rouillé et al., 2006; Ripoli et al., 2010; Horner et al., 2011). The ER-mitochondria contact sites, also known as mitochondria associated membranes (MAMs) (Mannella et al., 1998; Rizzuto et al., 1998), are a well-organized intracellular synapse-like inter-organelle communicating systems whose structural tethering components have been elucidated (Raturi and Simmen, 2013; Giorgi et al., 2015; Giacomello and Pellegrini, 2016). The main proposed function of MAMs is to provide a tightly controlled, localized flux of calcium from the ER store into mitochondria without raising its concentration in the cytosol (Rizzuto and Pozzan, 2006; Krols et al., 2016). Calcium is a recognized physiological modulator of the mitochondrial metabolism, though above a threshold level it becomes cytotoxic (Duchen, 2000).

On this background, we have investigated functional properties of mitochondria in the context of HCV infection. To this aim we used two well-established *in vitro* cell models. One is a tetracycline-regulated system allowing the inducible expression of the entire HCV polyprotein or of defined parts thereof in stably transfected U-2 OS human osteosarcoma cells (Moradpour et al., 1998); in the inducible system only transcription and translation of the viral proteins occurs. The other is an infective system where the virus accomplishes its entire life cycle in the permissive HCC-derived cell line Huh-7.5; to track infected cells GFP was inserted into the HCV genome (Moradpour et al., 2004; Schaller et al., 2007).

**Figure 1:**
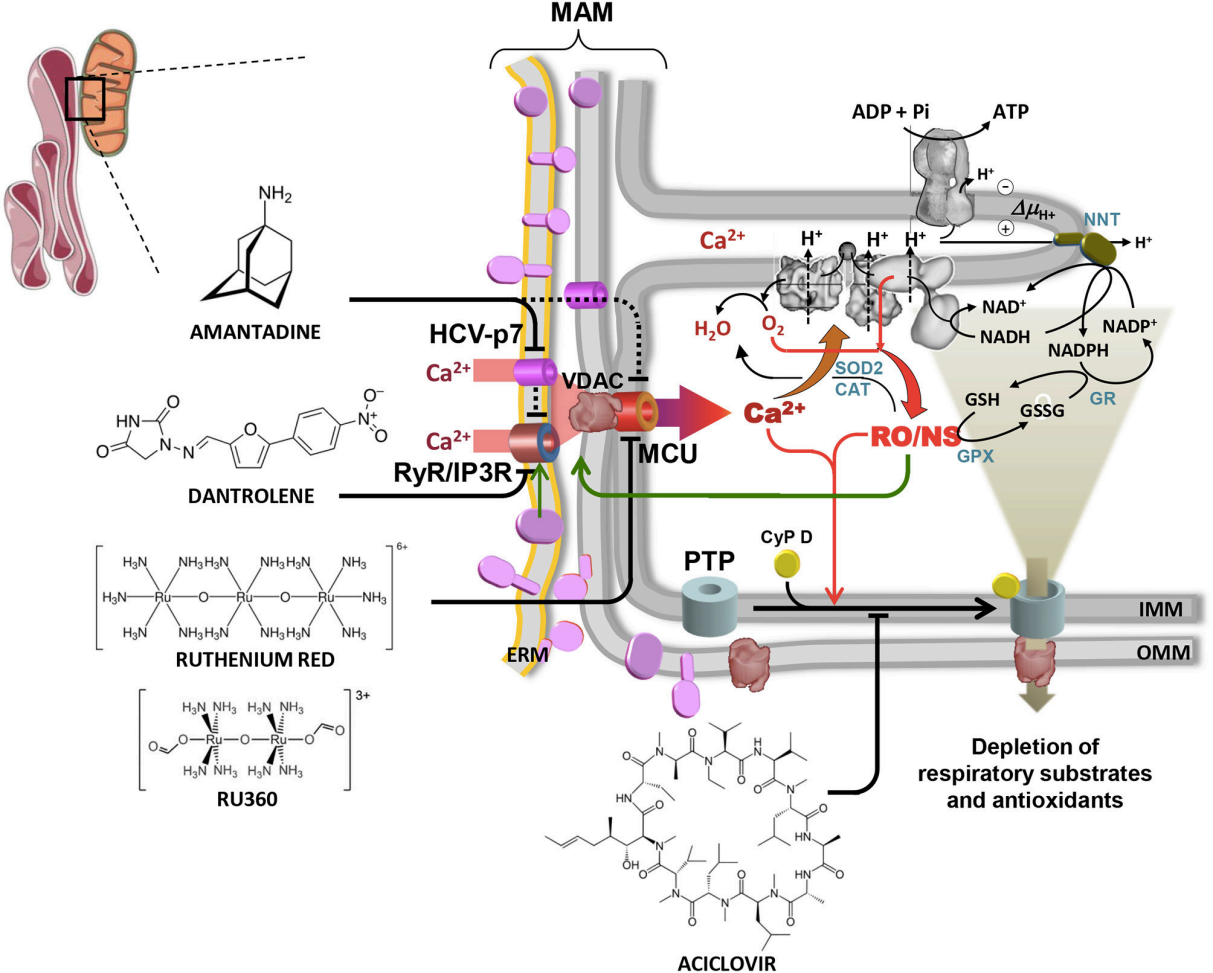
Overview of the alterations in mitochondrial physiology linked to HCV protein expression and of drug-targetable ion channels and translocators. The contact site between endoplasmic reticulum and mitochondria is shown (mitochondrial associated membrane, MAM). HCV proteins are shown in purple on the ER membrane (ERM) and on the outer mitochondrial membrane (OMM) where they are likely transferred from ERM by lateral diffusion. Interaction of HCV proteins with the ERM-located Ca^2+^ transporters (RyR/IP3R) is shown, causing localized increase of the [Ca^2+^], thereby promoting its uptake within the mitochondria via the voltage-dependent anion channel (VDAC) and the mitochondrial calcium uniporter (MCU) located in the OMM and IMM respectively. The viroporin HCV p7 is also shown to contribute to the mitochondrial Ca^2+^ load. Activation of the ER unfolded protein response (UPR) linked to the stressing accumulation of HCV proteins might also be indirectly involved in the deregulation of the inter-organelle Ca^2+^ fluxes (not shown). The increased intramitochondrial [Ca^2+^] affects components of the respiratory chain (RC) and activates mitochondrial nitric oxide synthase (not shown, but see text), thereby increasing generation of reactive oxygen and nitrogen species (RO/NS). RC is illustrated within the IMM cristae with complexes I, III, IV (from right to left). The F_o_F_1_ H^+^-ATP synthase is also shown in the upper part of the crista. Electron transfer from NADH to O_2_ (forming H_2_O) and the chemiosmotic circuitry coupling RC proton pumping to the ΔΨ-driven ATP synthesis are shown by black arrowed lines. Electron leak from dysfunctional RC leads to ROS formation/accumulation (red arrowed lines) which is mitigated by the concerted action of Mn-superoxidase dismutase 2 (SOD2), catalase (CAT) and glutathione peroxidase (GPX) with glutathione reductase (GR) and nicotinamide nucleotide transhydrogenase (NNT) needed to regenerate reduced glutathione (GSH) at expense of NADPH. The enhanced intramitochondrial levels of Ca^2+^ and RO/NS are shown to activate the permeability transition pore (PTP), illustrated schematically by the cyclophilin D (CyP D)-mediated assembly of OMM and IMM components. Opening of the PTP causes flush out of low molecular weight metabolites comprising NAD(P)^+^/NAD(P)H and GSH/GSSG. This leads to further impairment of the RC activity (and of the oxidative phosphorylation) and to reduced ROS scavenging with consequent worsening of the redox balance. RO/NS are finally shown to affect the activity of the Ca^2+^ transporting system (green arrow) with further entry of Ca^2+^ into the mitochondria, thereby fuelling a positive feedback mechanism. Blocking the above-illustrated cycle of alterations constitutes a rationale to develop therapeutic strategies. Accordingly, inhibitors of the ER Ca^2+^ channel(s) (dantrolene), the viroporin p7 (amantadine), the MCU (ruthenium red/Ru360), the PTP (aciclovir) are shown. Reproduced with modifications from Quarato et al. (2013). Copyright (2013), with permission from Elsevier.

The main results of systematic studies carried over the past decade by our group are schematically illustrated in Figure 1 (Piccoli et al., 2007; Ripoli et al., 2010; Quarato et al., 2012, 2014). It is shown that expression of the HCV proteins both in the inducible system and in Huh 7.5 cells transfected with infectious full-length HCV leads to profound alterations of the mitochondrial functions. These comprise: (i) intra-mitochondrial calcium (mtCa^2+^) overload; (ii) dissipation of the mitochondrial membrane potential (ΔΨ_m_), which correlates with inhibition of cell respiration and complex I (NADH dehydrogenase) activity; (iii) overproduction of reactive oxygen and nitrogen species (RO/NS). Time-resolved analysis demonstrated that mtCa^2+^ overload was the earliest mitochondria-related alteration following induction of HCV protein expression (Piccoli et al., 2007; Quarato et al., 2012).

## Inhibitors of the intra-mitochondrial Ca^2+^ flux dampen HCV-mediated mitochondrial dysfunction

The major transporter of Ca^2+^ into mitochondria is the mitochondrial calcium uniporter (MCU) (De Stefani et al., 2011). MCU is part of a complex, comprising also regulatory subunits, mediating a ΔΨ_m_-driven accumulation of calcium ions on the negative site of the inner mitochondrial membrane (Marchi and Pinton, 2014; Granatiero et al., 2017). From the kinetic point of view, MCU is a very low-affinity, high-capacity transporter, meaning that though possessing a relatively high Km for Ca^2+^ its abundance in the inner mitochondrial membranes makes mitochondria an efficient buffering compartment preventing harmful Ca^2+^ rising in the cytosol (Deryabina et al., 2004). The kinetic limitation of MCU is overcome by MAMs, which releasing ER-calcium in its intermembrane space provides a relatively high-concentration Ca^2+^ microdomain (Rizzuto and Pozzan, 2006). The ER possesses a number of calcium releasing channels comprising inositol trisphosphate (IP3) and ryanodine receptors (Patterson et al., 2004; Hamilton, 2005).

Importantly, when cells inducibly expressing the HCV polyprotein were treated with ruthenium red (RR) or Ru360, both inhibitors of the MCU (Broekemeier et al., 1994; Matlib et al., 1998), the above-reported mitochondrial alterations were fully prevented (Piccoli et al., 2007). A similar protection was observed when these cells were treated with dantrolene, an inhibitor of the ER calcium channels (Piccoli et al., 2007).

Taken together, the results obtained led us to suggest a working model whereby the overload of mtCa^2+^ is the seminal event in the successive alterations (Piccoli et al., 2006, 2009; Quarato et al., 2013). Possibly, overcrowding of HCV proteins at MAMs might affect the overall calcium retention capacity of the ER membranes as a consequence of a mild unfolded protein response (UPR) (Carreras-Sureda et al., 2017) or elicit a specific effect on the Ca^2+^ channels (Deniaud et al., 2008). The MCU-mediated load of Ca^2+^ into the mitochondria drives alterations in the redox homeostasis. This might be achieved by activation of a Ca^2+^-dependent mitochondrial isoform of the nitric oxide synthase (Dedkova et al., 2004; Ghafourifar and Sen, 2007). NO is known to affect the mitochondrial respiratory chain by competitive inhibition of the cytochrome c oxidase and/or by covalent modification of complex I (Brown and Borutaite, 2004; Sarti et al., 2012). Impairment of the normal electron transfer in the respiratory chain results in enhanced electron leak to O_2_, with formation of the superoxide anion (${\text{O}}_{2}^{•-}$) (Murphy, 2009), which is further converted to H_2_O_2_ by the Mn-SOD. To support this model is the evidence provided by confocal microscopy analysis, using specific probes for NO, ${\text{O}}_{2}^{•-}$, and peroxides, showing a clear compartmentalization of the fluorescent signals resembling the mitochondrial network (Piccoli et al., 2007). Overproduction of ROS has been recurrently reported to enhance mtCa^2+^ uptake likely by modification of redox sensitive cysteines of the ER calcium channels (Feissner et al., 2009; Görlach et al., 2015) and/or of the MCU (Dong et al., 2017). Accordingly, treatment of cells inducibly expressing the HCV polyprotein with the antioxidant N-acetylcysteine (NAC) prevented completely the mtCa^2+^ overload as well as inhibition of the mitochondrial respiratory activity and of the ΔΨ_m_ generation (Piccoli et al., 2007).

The enhanced levels of Ca^2+^ and RO/NS into mitochondria proved to activate a long established feature of the organelle better known as mitochondria permeability transition (MPT) (Giorgio et al., 2017). MPT consists in increased non-specific conductance of the inner mitochondrial membrane to low molecular weight molecules (<1,500 Da). Activation of the MPT is attained by a number of factors promoting binding of cyclophilin D (Cyp D) to the MPT pore (Elrod and Molkentin, 2013). The molecular nature of the MPT pore has been elusive for a long time though recent evidence suggests the F_o_F_1_-ATP synthase as a plausible candidate (Bernardi et al., 2015; Jonas et al., 2015). Transient opening (i.e., flickering) of the MPT pore works as a relief valve-like system avoiding hyperpolarization of the inner mitochondrial membrane as well as recycling of Ca^2+^ (Aon et al., 2008; Nivala et al., 2011). Conversely, permanent opening of the MPT pore causes exit of low molecular weight antioxidants (like glutathione) and redox coenzymes fostering oxidative stress as well as swelling of mitochondria because of its hyperosmolarity as compared with the cytosol (Di Lisa et al., 2001). This event can lead to autophagy of the organelle, apoptosis or necrosis depending on the prevailing cellular setting (Kroemer et al., 2007).

To verify the involvement of the MPT in the observed HCV-mediated mitochondrial dysfunctions we tested the effect of alisporivir, a robust antiviral drug (Paeshuyse et al., 2006; Coelmont et al., 2009; Gallay and Lin, 2013). Alisporivir is a cyclosporin A analog but without immunosuppressive properties (Gallay and Lin, 2013). It binds to Cyp D, interfering with its opener function of the MPT (Elrod and Molkentin, 2013). When alisporivir was tested on the mitochondrial dysfunctions caused by HCV protein expression we found an impressive capability of the drug to fully prevent (and even reverse) the ΔΨ_m_ collapse, RO/NS production and mtCa^2+^ overload (Quarato et al., 2012).

Combination of all the above reported observations supports a pathogenetic model for HCV infection whereby a self-nourishing mechanism is activated, consisting in positive feed-back loops initiated by the entry of Ca^2+^ into mitochondria and fuelled by the ensuing RO/NS overproduction elicited by impaired activity of the respiratory chain (Figure 1). In such a cascade of events an essential role is seemingly played by mitochondrial and ER transporters (i.e., MCU, MPT pore, ER-Ca^2+^ channels) since inhibition of either of them prevents and reverses the HCV protein-mediated mitochondrial alterations.

## Exploring the HCV viroporin p7 as a potential therapeutic target

The impact of Ca^2+^ flux homeostasis in the interplay between HCV and the host cell is also underlined by the presence in the HCV proteins of the viroporin p7 (Madan and Bartenschlager, 2015). p7 is a transmembrane protein constituted by two transmembrane helices which is thought to oligomerize in hexameric structures, forming a channel (Clarke et al., 2006). When inserted into artificial membranes, p7 proved to increase ionic conductance with selectivity toward cations (Griffin et al., 2003; Montserret et al., 2010; Wozniak et al., 2010). It accumulates in ER membranes and is particularly enriched at the MAMs sub-compartment (Griffin et al., 2004). Data obtained *in vitro* suggested a role of the antiviral drug amantadine in inhibiting HCV p7-mediated cation conductance (Griffin et al., 2003; Cook et al., 2013; see also Atoom et al., 2014). Given this premise and in keeping the observed mitochondrial alterations caused by HCV protein expression we tested on those the effects of amantadine, an adamantane-derived compound (Figure 1). We found that amantadine not only prevented but also rescued HCV protein-mediated mitochondrial dysfunction in cells inducibly expressing the HCV polyprotein (Quarato et al., 2014). Specifically, amantadine corrected: (i) overload of mitochondrial Ca^2+^; (ii) inhibition of respiratory chain activity and oxidative phosphorylation; (iii) reduction of membrane potential; (iv) overproduction of reactive oxygen species. The effects of amantadine were observed within 15 min following drug administration and confirmed in Huh-7.5 cells transfected with the infectious full-length HCV genome. However, these effects were also observed in cells expressing subgenomic HCV constructs, indicating that they are not mediated or only in part mediated by p7. Single organelle analyses carried out on isolated mouse liver mitochondria demonstrated that amantadine induces hyperpolarization of the membrane potential (Quarato et al., 2014). Moreover, amantadine treatment increased the calcium threshold required to trigger mitochondrial permeability transition opening (Quarato et al., 2014). These results led to the conclusion that amantadine displays off-target effects likely relatable to Ca^2+^ transporting systems of the host cell.

## Metabolic rewiring of host cell by HCV

Surprisingly, in spite of the overt impairment of the mitochondrial respiratory chain activity and of the concurrent oxidative phosphorylation (OxPhos), HCV-infected cells did not show evident signs of sufferance or bioenergetic failure. Indeed, the difference in growth rate and viability of both HCV-induced U2-OS cells and HCV-infected Huh-7 cells was negligible as compared with control cells. Accordingly, the cellular ATP level was unaffected, if not increased, in HCV-induced U2-OS cells grown in glucose-containing media (Piccoli et al., 2007). Though this is consistent with the non-cytopathic property of HCV, it implies the need to understand how the virus rewires the host cell metabolism (Diamond et al., 2010).

**Figure 2:**
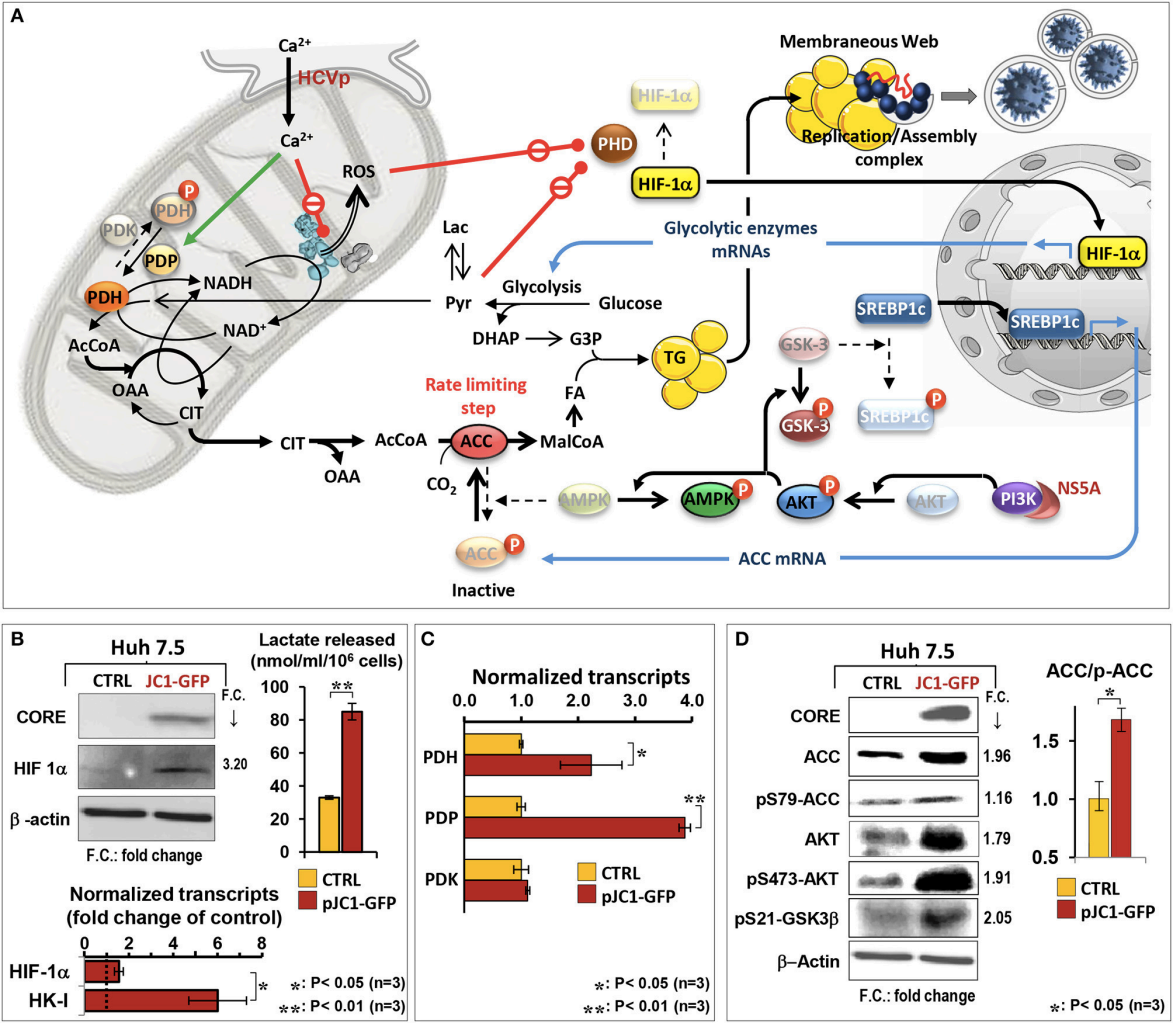
HCV induces rewiring of cell metabolism in infected cells. **(A)** The scheme summarizes major changes in the metabolic pathways induced by HCV infection as supported by evidence reported in the literature and by the unpublished results showed in panels **(B–D)** obtained in HCV Jc1 RNA-transfected Huh-7.5 cells *in vitro*. HCV protein-induced enhanced entry of Ca^2+^ into mitochondria is shown to dampen the respiratory chain activity and oxidative phosphorylation (OxPhos) and to elicit increased reactive oxygen species (ROS) production. These inhibit the prolyl-hydroxylase (PHD) leading to stabilization of the hypoxia induced transcription factor (HIF-1α) which controls the expression of the glycolytic enzymes, thereby shifting cell metabolism toward aerobic glycolysis. **(B)** shows the stabilization of HIF-1α and the consequent metabolic shift evidenced by upregulation of the hexokinase I (HK-I) transcript and by increased lactate release in transfected Huh-7.5 cells. The enhanced glycolytic flux leads to accumulation of pyruvate which proved to further inhibit PHD. Pyruvate enters into mitochondria where it is converted in acetyl-CoA (AcCoA) by the pyruvate dehydrogenase (PDH). The HCV protein-mediated load of Ca^2+^ into mitochondria is shown to activate the pyruvate dehydrogenase phosphate (PDP), which controls the activity of the PDH. To note, at the transcriptional level both PDH and PDP [but not the pyruvate dehydrogenase kinase (PDK)] are significantly up-regulated in HCV RNA-transfected Huh-7.5 cells **(C)**. The enhanced production of AcCoA leads to formation of citrate (CIT), which because of the limited availability of oxidized NAD^+^ (caused by impaired respiratory chain activity) is not further transformed *via* the tricarboxylic cycle and exits from mitochondria to shuttle AcCoA in the cytosol. The cytosolic AcCoA functions as precursor for the *de novo* synthesis of fatty acids (FA) that with intermediates of glycolysis forms triglycerides (TG) accumulating as lipid droplets. **(D)** shows that HCV RNA-transfected Huh-7.5 cells displays a two-fold increased expression of the acetyl CoA carboxylase (ACC), the controlling step in FA synthesis. ACC activity is controlled by its inactivating phosphorylation mediated by the AMP-activated protein kinase (AMPK) which is in turn controlled by the phosphorylated state of Akt/protein kinase B. Phosphorylation of AKT is mediated by activation of the phosphatidylinositol 3-kinase (PI3K), which has been reported to interact with HCV NS5A. Notably, the phosphorylated AKT is known to inactivate the glycogen synthase kinase 3β (GSK3β) which inhibits the activity of the transcription factor sterol regulatory element-binding protein 1c (SREBP 1c) controlling the expression of ACC. Consistently, the Western blots in panel **(D)** show enhanced phosphorylation of both AKT and GSK3β. The inability of HCV RNA-transfected to properly oxidize FA by the mitochondrial β-oxidation (requiring efficient respiratory chain) may lead to cytosolic accumulation of acyl-CoA which flow into TG synthesis (not shown). Lipid droplets results in formation of a membraneous web, contributed also by the HCV protein induced extensive rearrangement of host cell membranes, which contains the sites of viral replication and possibly assembly.

An important factor and regulator of metabolism in cells challenged by stressing conditions is constituted by hypoxia inducible factor 1α (HIF-1α) (Wang and Semenza, 1993). HIF-1α is rapidly degraded under normal oxygen tension following hydroxylation by O_2_- and 2-oxo-glutarate-dependent prolyl hydroxylases (PDHs) (Bruick and McKnight, 2001). The hydroxylated HIF-1α is then ubiquitinated and steered toward proteasomal degradation (Mole et al., 2001). Conversely, under hypoxic conditions the hydroxylation of HIF-1α is dampened and it accumulates and moves to the nucleus where promotes transcription of a number of prosurvival genes, including those coding for glycolytic enzymes (Semenza et al., 1994; Wang et al., 1995). However, conditions different from hypoxia, which in turn inhibit PDH activity (i.e., RO/NS and/or competing 2-oxo-acids) result in stabilization of HIF-1α also under normoxia (Déry et al., 2005; Pugh, 2016; Figure 2A).

We demonstrated that HIF-1α is stabilized under normoxic conditions both in HCV-induced U2-OS and HCV-infected Huh-7 cells as well as in patients' liver biopsies (Ripoli et al., 2010; see also Nasimuzzaman et al., 2007; Wilson et al., 2012). Consistent with this finding, we showed that the HIF target genes coding for the glycolytic enzymes hexokinases I and II (HKI, HKII) were both upregulated at the transcriptional and protein levels. This would indicate a metabolic shift toward aerobic glycolysis that we supported by an observed higher release of lactate in HCV-induced U2-OS cells (Figure 2B). It is worth considering that HKII was shown to interact with the outer mitochondrial membrane at the level of the voltage-dependent anion channel (VDAC) and to prevent MPT activation (Pastorino et al., 2005; Chiara et al., 2008). Also, HKI was found to interact with the outer mitochondrial membrane, thereby blocking apoptotic signals (Abu-Hamad et al., 2008; Schindler and Foley, 2013).

On this basis, we proposed a model, consistent with other reported evidence, whereby the MPT pore oscillates between the closed and open state under the positive influence of HKII, which restrains the effects of RO/NS and mtCa^2+^ (Quarato et al., 2012). Stabilization of HIF-1α, up-regulating the expression of HKII, is likely to be linked to activation of the AKT-mTOR pathway (Land and Tee, 2007; Agani and Jiang, 2013). Indeed it has been suggested that PI3K, the upstream activator of AKT, binds the HCV NS5A protein which activates it permanently (Street et al., 2005). Moreover, the active form of AKT deactivates GSK-3β, which by phosphorylation of VDAC displaces HKII, promoting permanent opening of the MPTP under stressing conditions (Pastorino et al., 2005; see also Chiara et al., 2008). In agreement with this proposal, we found that the phosphorylation state of AKT and GSK-3β was enhanced both in HCV-induced U2-OS and in HCV-transfected Huh 7.5 cells (Figure 2D).

We have here a remarkable example of the strategy put in action by HCV which although impairing the most important energetic powerhouse of the cell (i.e., the mitochondrial OxPhos system) at the same time tunes the consequent effects evading premature cell-death signaling of the host cell.

## Inhibitors of the ER and mitochondrial Ca^2+^ channel/porter damper lipogenesis in HCV infected cells

A recently emerged property of HCV is the enhanced mitophagy in the host cell (Kim et al., 2013, 2014; Ruggieri et al., 2014). Mitophagy is a selective autophagic degradation of mitochondria which is part of a quality control processing of the cell (Anding and Baehrecke, 2017). The main trigger for recognizing damaged mitochondria is a drop in the membrane potential which recruits and activates mitophagic factors like PINK1 and parkin to initiate first the fission of the mitochondrial network and then the engulfment of the isolated mitochondria into isolation membranes to become mitophagosomes (Zimmermann and Reichert, 2017). However, and remarkably in the context of HCV infection, this process appears to be somehow abortive since the mitophagosomes, instead of fusing with lysosomes, have been reported to accumulate, together with lipid droplets, in the membranous web which is the peculiar environment where HCV RNA replication takes place (Hara et al., 2014). Blocking this process results in suppression of the viral replication (Fang et al., 2017). This is possibly another strategy to hinder the MAVS-mediated immune response.

**Figure 3:**
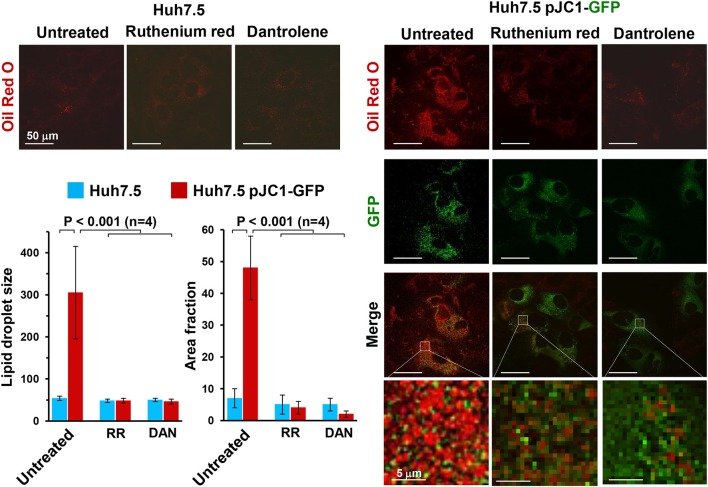
Effect of ruthenium red and dantrolene on lipid droplet formation in transiently HCV RNA-transfected Huh-7.5 cells. Lipid droplets were stained with Oil Red O (3 mg/ml for 60 min) and imaged by laser scanning confocal microscopy. Representative images of control and Jc1-GFP RNA-transfected Huh-7.5 cells are shown illustrating the merge of Oil Red O (red) and GFP-related (green) fluorescence signals; the latter was used to track HCV-transfected cells (>90% of the cell population). Control Huh-7.5 cells were subjected to the transfection protocol but without HCV RNA. The time point after transfection was 72 h (see Ripoli et al., 2010 for further details). Where indicated 5 μM Ruthenium red (RR) or 10 μM dantrolene (DAN) were added soon after transfection. Enlargements of the merged pictures are also shown to better visualize lipid droplets in HCV RNA-transfected Huh-7.5 cells. The histograms of the Oil Red O-related fluorescence in GFP-positive transfected cells comparing untreated and drug-untreated cells are shown and refer to the averaged size and to the occupied cellular area fraction of the lipid droplets. The fluorescence intensity was assessed by averaging 10–20 cells from each of at least 10 optical fields under each condition using ImageJ 1.49v (http://imagej.nih.gov/ij). The average of four independent experiments plus the standard error of the mean (SEM), along with statistical analysis, is shown.

Accumulation of lipid droplets can be easily detected by staining cells with Oil Red O. Figure 3 shows the impressive accumulation of lipid droplets in HCV-infected Huh 7.5 cells and the co-localization of them with GFP-labeled NS5A HCV protein. Similar results were obtained with HCV-induced U2-OS cells. This observation clearly implies an HCV-mediated deregulation of fatty acid metabolism, which may derive from an inhibition of fatty acid oxidation (FAO) or increased fatty acid synthesis (FAS) or both. Deregulation of peroxisome proliferator-activated receptors (PPARs), the master transcription factors regulating lipid metabolism, is likely to be involved (Agriesti et al., 2012). Notably, treatment of infected cells with either dantrolene or RR prevented lipid droplet accumulation, pointing once again to alteration of the ER-mitochondria calcium flux as a germinal event in HCV infection.

Hampering of FAO can be envisioned as a consequence of the HCV-mediated impairment of respiratory chain function which limits the proper redox recycling of the coenzymes required in fatty acid β-oxidation. In addition, flickering of the MPT would result in progressive leakage from the mitochondrial compartment of factors needed for FAO (like carnitine) (Di Lisa et al., 2001).

However, also an enhanced fatty acid and triglyceride (TG) biosynthesis is likely to occur concurrently with FAO dampening. Consistently, we found up-regulation of the FAS rate-limiting enzyme acetyl CoA carboxylase (Wakil et al., 1983; Kim, 1997) both in terms of enhanced expression and of post-translational activating phosphorylation *via* the PI3K-AKT axis (Hardie, 1989; Figure 2D). Activation of the transcription factor SREBP1c, controlling the expression of ACC, is known to be linked to the AKT-mediated inactivation of GSK-3β (Kim et al., 2004; Park et al., 2009; Yecies et al., 2011). Such a signaling pathway was recently found to upregulate HCV RNA translation (Shi et al., 2016). Precursors for FAS and TG are provided by glycolytic metabolites. Acetyl CoA is largely derived by pyruvate oxidative decarboxylation, catalyzed by the pyruvate dehydrogenase (PDH) complex, and *via* the citrate shuttle released in the cytoplasm. The PDH function is tightly controlled by its phosphorylation state, which in turn depends on the balanced activities of a PDH kinase (PDK) and a PDH phosphatase (PDP) (Roche et al., 2001). To note, we found that HCV full-lenght RNA-transfected Huh 7.5 cells displayed enhanced transcript levels of PDH and PDP (Figure 2C). Remarkably, mtCa^2+^ is required to stimulate the PDH phosphatase and consequently the PDH, which is more active in its dephosphorylated state (Huang et al., 1998; Denton, 2009). This notion would explain the observed inhibition of lipid droplet accumulation in HCV-infected cells when treated with inhibitors of either the MCU or ER-calcium channel(s).

## Conclusions

**Figure 4:**
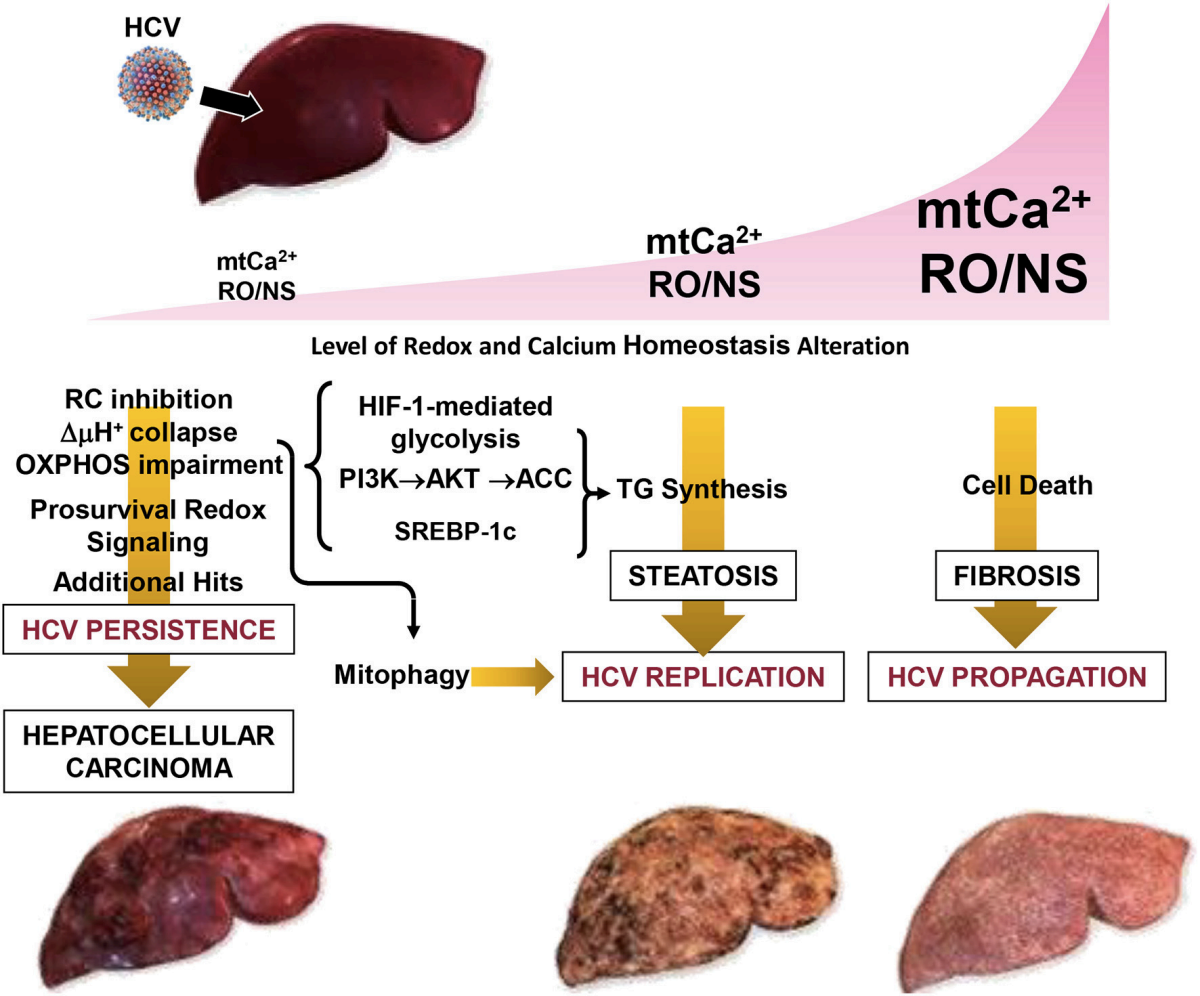
Pathogenetic model of HCV-mediated alterations. The development of disease in patients with chronic hepatitis C is modeled as a function of the level of the oxidative alteration and of the mt-Ca^2+^ load. Three pathogenic settings are presented. Low RO/NS- and mt-Ca^2+^-dependent stress level activates a pro-survival and proliferative adaptive response by redox signaling. The flickering balance between the mitochondrial PTP closed/open configuration is set to a level that causes collapse of the respiratory chain-mediated protonmotive force (i.e., ΔμH^+^), with consequent impairment of ATP synthesis by the FoF1-H^+^ ATP synthase. This forces the infected cell to shift its energy-supplying metabolism toward glycolysis by activation of the transcription factor HIF-1α. Collapse of the ΔμH^+^ is a trigger for selective removal of damaged mitochondria by the organelle-specific autophagic machinery (i.e., mitophagy), which is required for HCV replication. Such a prosurvival setting in the host cell facilitates HCV persistence. However, if additional (mutagenic) hits accumulate over the time this may result in clonal expansion, leading to hepatocellular carcinoma. Intermediate levels of RO/NS and mt-Ca^2+^ enhance the closed to open transition of the PTP causing, among others, depletion of low-molecular weight metabolites (i.e., glutathione, NAD^+^, carnitine, coenzyme A) needed to guarantee antioxidant capacity and import of long chain acyl-CoA (AcCoA) for β-oxidation. Accumulation of acyl-CoA leads to conversion into triglycerides (TG). Other factors, described in Figure 2 (i.e., activation of HIF-1α, PI3K-Akt-ACC) and of the sterol regulatory element-binding protein (SREBP-1c) may contribute to enhanced *de novo* lipogenesis. All together this may account for the steatosis which can be observed in HCV-infected hepatocytes. Accumulation of lipid droplets in the cytoplasm is believed to provide an assembly platform for HCV. High intramitochondrial concentrations of Ca^2+^ and ROS induces permanent opening of the PTP causing osmotic swelling and rupture of the outer mitochondria membrane. The consequent release of cytochrome c and other pro-apoptotic factors triggers the caspase cascade. Depending on the intracellular ATP level, this would lead to apoptosis or necrosis activating, in the last case, tissue fibrosis. Reproduced with modifications from Quarato et al. (2013), Copyright (2013), with permission from Elsevier.

In conclusion, the emerging strategy put in action by HCV is consistent with a fine rewiring of host cell metabolism. This is achieved by depressing mitochondrial OxPhos while fostering glycolysis which provides, in addition to energy, precursors for biosynthetic processes. A major and perhaps germinal event appears to be a deregulation of Ca^2+^ flux homeostasis between the ER and mitochondria at specialized contact sites. This would selectively target mitochondria, avoiding large changes of the Ca^2+^ concentration in the cytosol, thus preserving cell viability. The ensued increase of mtCa^2+^ leads to changes in the mitochondrial redox tone. This results in progressive dysfunction of the respiratory chain activity and the resulting OxPhos failure is bioenergetically compensated by an enhanced glycolytic flux. In this context, concurrent or consequent activation of prosurvival transcription factors contributes to dampen cell death. Realization of a membranous web constituted by accumulation of lipid droplets, and possibly by incomplete mitophagy, provides a suitable platform for HCV replication and virus particles assembly. Depending on the prevailing conditions (i.e., the level of the oxidative alterations and of the mtCa^2+^ load) in such a multistep process, HCV infection can progress to different clinical outcomes including steatosis, fibrosis, HCC (Figure 4).

Importantly, the development of drugs selectively targeting ER and/or mitochondrial calcium channels might represent a potential strategy in support of standard HCV therapies.

## Author contributions

RS: Carried out the experiments, analyzed the results; CP: Designed and supervised the study, carried out the experiments; DM: Provided expertize, samples and critical feedback, assisted in writing the paper; NC: Designed the study, supervised the project, wrote the paper.

### Conflict of interest statement

The authors declare that the research was conducted in the absence of any commercial or financial relationships that could be construed as a potential conflict of interest.
